# Enhancing Science Communication through Deliberative Discussions on Water Contamination in a General Chemistry Lab at an HBCU

**DOI:** 10.1021/acs.jchemed.5c01446

**Published:** 2026-07-21

**Authors:** Navneet Goyal, Margo N. Montgomery-Richardson, Abha Verma, Siddieg O Elsiddieg, Jayalakshmi Sridhar

**Affiliations:** Department of Chemistry, 5785 Xavier University of Louisiana, New Orleans, Louisiana 70124, United States

**Keywords:** Undergraduate, General chemistry laboratory, Water contamination, Policy

## Abstract

In Spring 2023 (pilot study, n = 24) and Spring 2024 (main study, n = 48; survey respondents n = 36), second-semester General Chemistry II laboratory students at Xavier University of Louisiana, a Historically Black College and University (HBCU), participated in a classroom activity designed to enhance science communication skills through deliberative discussions on water contamination issues. Following qualitative analysis experiments on heavy metal ions, students engaged in structured small-group deliberations evaluating real-world water contamination challenges and policy solutions. The activity was explicitly aligned with the United Nations Sustainable Development Goal (SDG) #6: Clean Water and Sanitation, providing a global sustainability framework for student discussions. Survey results indicate increased student engagement, improved understanding of water contamination, and enhanced confidence in communicating scientific concepts to nonspecialist audiences. While most students reported increased interest in science communication and STEM careers, some indicated the need for additional support to build confidence. These findings demonstrate that integrating deliberative, sustainability-focused discussions into laboratory courses effectively connects experimental chemistry to societal challenges while fostering scientific literacy and communication skills.

## Graphical abstract

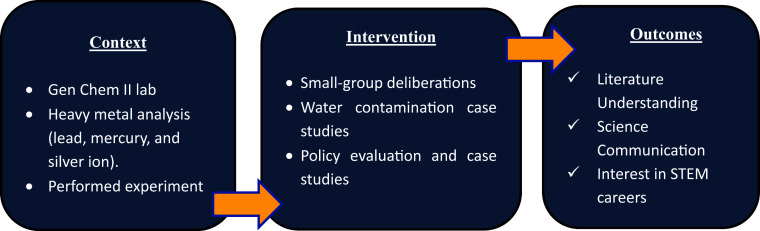

## Introduction

Effective science communication plays a critical role in ensuring accurate data collection, promoting safety, and advancing scientific understanding in chemistry education and laboratory practices.^1,2^ However, it is often neglected, and students may lack the necessary soft skills to communicate their ideas effectively with peers and the larger community.^1–5^

Several studies have emphasized the importance of science communication to varied audiences in chemistry education.^6^ The National Research Council (NRC) has identified communication as one of the five core competencies for undergraduate chemistry students, and the American Chemical Society (ACS) has emphasized the significance of effective communication skills in the chemistry profession.^7^ Research has also shown that students who are trained in science communication are more successful in their careers and better equipped to communicate their research findings to a broader audience.^7^

Despite the emphasis on science communication, many students struggle to communicate their ideas effectively due to several communication obstacles in the laboratory setting such as language barriers, cultural differences, lack of confidence, and lack of training in science communication.^8^ Additionally, students may lack opportunities to practice and refine their communication skills, especially in the context of group work and collaborative projects.^9^

Efforts have been made to improve both written and oral communication skills in chemistry courses; however, the literature on chemical communication pedagogy remains notably limited in introductory-level chemistry lab courses.^10,11^ Poster presentations have been utilized for various purposes, including promoting the study of chemistry, facilitating student learning, generating enthusiasm, and serving as assessment tools.^12–14^ In addition to the poster approach, another group introduced an instructional model called Argument-Driven Inquiry in their general chemistry lab.^15^ This model involved incorporating a double-blind group peer review system for student laboratory reports, enabling students to revise their reports based on the feedback received.^15^

Our approach aimed to encourage students to reflect on their experiments, establish connections with real-life applications, and explore ways to effectively communicate their findings to peers and society. We achieved this by emphasizing a deliberative process and focusing on oral communication to enhance their knowledge and skills in chemistry communication.

## Water Contamination Issues

Access to clean drinking water is considered an essential part of life. Two of the primary water concerns in the United States are deteriorating water infrastructure and a lack of financing.^16^ Because of residential segregation in the United States, populations of color and low-income groups are more likely to have water infrastructure that is outdated, underdeveloped, and underfunded.^16^ Water systems that are underfunded usually result in multiple violations with regards to monitoring the system for known contaminants. According to a report by the National Resource Defense Council, there was a higher likelihood of drinking water infractions of the Safe Drinking Water Act, long-term noncompliance, and low enforcement in counties with higher racial, housing, transportation, and economic mobility. This study explicitly connects laboratory instruction with the United Nations Sustainable Development Goal (SDG) #6: Clean Water and Sanitation, highlighting inequities in water infrastructure and access, particularly in marginalized communities.^16^

Studies have shown that New Orleans’ water supply is susceptible to contamination by metals and polycyclic aromatic hydrocarbons (PAHs).^17,18^ In 2013, a study reported that some of the city’s water sources contained elevated levels of lead and copper.^17^ Another study published in 2016 found detectable levels of PAHs in wastewater samples from the area.^18^ The presence of metals and PAHs in water sources can have significantly negative impacts on human health and the environment. Therefore, it is essential to continue monitoring and analyzing water quality in the region to ensure that the water is safe for consumption and does not pose risks to public health. This work addresses a critical gap in introductory chemistry education by embedding structured deliberative discussions into laboratory instruction, enabling students to connect experimental observations with societal and policy contexts. We were particularly interested in this topic because it relates directly to an experiment we conduct in our General Chemistry teaching laboratory. Making connections between classroom learning and real-world issues reinforces the significance of the concepts students are studying and their practical applications.

### Learning Objectives

After completing this activity, students will be able to

- Explain qualitative analysis of Group I metal ions (Pb^2+^, Ag^+^, Hg_2_^2+^).
- Interpret experimental results in the context of environmental contamination.
- Communicate scientific findings effectively to nonspecialist audiences.
- Analyze water contamination issues through scientific and policy perspectives.
- Engage in evidence-based deliberation and collaborative decision-making.

## Deliberation Framework

Science communication, with the aim of sharing scientific knowledge with peers and the wider community, faces complexity due to ideology, sociology, and trust. Deliberative democracy^19^ is a two-way communication approach in which scientists translate knowledge into a useful form by considering the opinions of a diverse population for decision-making. Deliberation between science experts and the public enhances public awareness and democratic participation in science.^20^ These deliberations seek to find common ground and foster an exchange of ideas. Adequate training of scientists and science proponents in effective communication is crucial to the model of deliberative democracy.^21–23^

For this study, deliberations were conducted in small groups of 3–4 students, lasting approximately 30–40 min, facilitated by faculty and senior chemistry students. Prompts were provided regarding local water contamination challenges, and students were encouraged to reach consensus where possible.

## Participantion Demographic

The study was conducted in the Department of Chemistry at Xavier University of Louisiana (XULA), a Historically Black and Catholic University (HBCU) in New Orleans, USA. XULA’s mission is to create a more just and humane society by preparing its students to assume roles of leadership and service in a global context. The General Chemistry II laboratory (CHEM1021L) is offered to second-semester freshmen who have completed General Chemistry I (CHEM1011L). Several sections run simultaneously, each with a maximum of 24 students. During Spring 2023, 24 students in one section participated in this pilot study, while in Spring 2024 the study was expanded to two sections, with a total of 48 students enrolled. More than 95% of the students identified as belonging to underrepresented communities. Two additional faculty members and two senior chemistry majors assisted with facilitation to ensure smooth deliberation and provide guidance when necessary.

## Experiment

### Classroom Implementation Timeline

11-week course; pilot study during weeks 5–6.

#### Week 5 (Group-1-week-1)

Students performed qualitative analysis of Group I elements (Pb^2+^, Ag^+^, Hg_2_^2+^) in known solutions and were reminded to dispose of metals responsibly.

#### Week 6 (Group-1-week-2)

Students tested unknown solutions and prepared for deliberation. Prereadings on “forever chemicals” in New Orleans, floodwater contamination, and Flint water contamination were posted on D2L Brightspace.^24–26^

### Deliberation Activity

Students were divided into six groups, guided by faculty and senior students, discussing challenges related to clean drinking water. Prompts were provided, but students could form their own opinions.

### Video Assignment

Students were encouraged to create videos summarizing discussions and proposing solutions. Participation was voluntary; only a few students submitted videos (link in the Supporting Information (SI))

## Hazard Statement

Even though a very dilute solution of metals was provided, lead, mercury, and silver compounds are toxic and must be handled with appropriate personal protective equipment (PPE), including gloves and goggles. All waste was disposed of in a designated metal waste container. Students were explicitly instructed on safe handling and disposal procedures metal waste container. Students were explicitly instructed on safe handling and disposal procedures.

## Survey and Assessment

The survey was approved by the Institutional Review Board (IRB) at Xavier University of Louisiana (#965). Of the 48 students enrolled in two separate sections (24 students each) of the Spring 2024 General Chemistry Teaching Laboratory course, 36 students responded to the survey, which was completed within 1 week after the deliberation activity. In comparison, only 4 students participated in the survey during Spring 2023; therefore, the present analysis focuses on the Spring 2024 data. After the activity, students completed a 40-question survey (SI) using a 5-point Likert scale, divided into six sections. The survey collected information on students’ majors, qualitative feedback, and perceptions of science communication and deliberation.

## Results Summary

Results have been summarized, please see SI for more details.

### Student Experience and Qualitative Insights

Qualitative responses revealed recurring themes of “supportive faculty” and “engaging laboratory experiences”, suggesting that the instructional environment played a critical role in shaping positive student perceptions. At the same time, students identified challenges such as time constraints, limited resources, and financial barriers, indicating areas for future refinement. These findings highlight the importance of both instructional design and structural support in maximizing the effectiveness of active-learning interventions.

### Understanding of Water Contamination

Survey results indicate that the activity was effective in enhancing conceptual understanding. A large majority of students (25 out of 36) somewhat agreed or strongly agreed that participation in the activity improved their understanding of water contamination issues, with only a small minority (3 students) expressing disagreement. This suggests that integrating real-world environmental contexts with laboratory experiments can significantly reinforce content comprehension.

### Science Communication Skills

Student responses demonstrate measurable gains in perceived communication ability. Approximately 61% of students (22 out of 36) reported confidence in their ability to present scientific information to a lay audience. Similarly, 23 out of 36 students agreed that the activity improved their ability to communicate science to the public. However, 14 students reported only moderate or low confidence, indicating that while the activity is effective, additional scaffolding—such as repeated practice, peer feedback, or structured communication exercises—may be necessary to further strengthen these skills.

### Deliberation and Decision-Making

The deliberative component of the activity contributed to student engagement in evidence-based reasoning. Analysis of responses related to decision-making indicates that most students were able to form or refine their positions on water contamination issues following discussion. Only 5.6% of students (2 out of 36) strongly agreed that they remained unable to reach a decision after deliberation, while the majority expressed confidence in their ability to evaluate options. These findings suggest that deliberative discussion can effectively promote critical thinking and informed decision-making in a scientific context.

### Student Attitudes toward the Activity

Student perceptions of the activity were generally positive. A majority of respondents (23 out of 36) supported the inclusion of similar assignments in other science courses, reflecting recognition of the value of integrating societal and policy discussions into scientific learning. However, responses regarding comfort level were more mixed, with some students expressing uncertainty or discomfort with this nontraditional approach. This variability suggests that students may require clearer framing and support when engaging in interdisciplinary or discussion-based activities in traditionally technical courses.

### Career Interest and STEM Engagement

Survey responses also indicate strong interest in STEM careers, with approximately 56% of students reporting high or very high interest in chemistry-related careers. Analysis across majors revealed that Chemistry Pre-Med and Psychology Pre-Med students exhibited the highest levels of interest in science communication-related careers, **with an overall average interest score of** 3.28 out of 5. These findings suggest that integrating communication-focused activities into laboratory courses may be particularly effective in engaging students already inclined toward health and science professions.

## Conclusion

This study demonstrates that integrating deliberative discussions into a general chemistry laboratory, particularly within the context of water contamination and sustainability, can meaningfully enhance students’ conceptual understanding, science communication skills, and engagement with real-world issues. Conducted at an HBCU, this approach highlights the value of culturally relevant and societally grounded pedagogy in supporting underrepresented students in STEM. While students showed notable gains in confidence and communication, the findings also underscore the need for continued scaffolding and repeated practice to fully develop these competencies. Overall, this work supports the incorporation of structured, discussion-based, and context-driven learning strategies as an effective means to bridge experimental chemistry with societal challenges and to foster scientifically literate and engaged future professionals.

## Supplementary Material







## Data Availability

The data that support the findings of this study are available upon request from the corresponding author, Dr. Navneet Goyal, who can be reached at ngoyal@xula.edu.

## Notes

Video link (PDF)
Water contamination survey questions (PDF)
Results (PDF)
