# Supplementary material for: Enhancing Science Communication through Deliberative Discussions on Water Contamination in a General Chemistry Lab at an HBCU
Source: J Chem Educ. 2026 Jul 21;103(8):4690–4. doi: 10.1021/acs.jchemed.5c01446 (PMC13472131; doi:10.1021/acs.jchemed.5c01446)
Supplement: Supplementary file 1 [file ed5c01446_si_001.pdf]

Enhancing Science Communication through Deliberative Discussions on Water Contamination in a General Chemistry Lab at an HBCU

Navneet Goyal[1*], Margo N. Montgomery-Richardson[1], Abha Verma[1], Siddieg O Elsiddieg[1], and Jayalakshmi Sridhar[1]
Department of Chemistry, Xavier University of Louisiana, New Orleans, LA-70124, USA.

Keywords: Undergraduate, General chemistry laboratory, Water contamination, Policy

This video was prepared by Ms. Christina Davis as an mp4 file.

Dr. Goyal has uploaded it to the YouTube and made it private. It can only be accessed using the link. (https://youtu.be/MF7dZPcEDts)
