# Supplementary material for: Enhancing Science Communication through Deliberative Discussions on Water Contamination in a General Chemistry Lab at an HBCU
Source: J Chem Educ. 2026 Jul 21;103(8):4690–4. doi: 10.1021/acs.jchemed.5c01446 (PMC13472131; doi:10.1021/acs.jchemed.5c01446)
Supplement: Supplementary file 2 [file ed5c01446_si_002.pdf]

Enhancing Science Communication through Deliberative Discussions on Water Contamination in a General Chemistry Lab at an HBCU

Navneet Goyal[1*], Margo N. Montgomery-Richardson[1], Abha Verma[1], Siddieg O Elsiddieg[1], and Jayalakshmi Sridhar[1]

Department of Chemistry, Xavier University of Louisiana, New Orleans, LA-70124, USA.

Keywords: Undergraduate, General chemistry laboratory, Water contamination, Policy

Water Contamination Survey Questions:

Section 1: Prior Awareness of Water Contamination Issues

You recently received three articles related to water contamination and its effects on communities in Michigan and Louisiana. Please indicate your level of agreement with the following statements:

1.1 Prior to reading the articles on water contamination in New Orleans, I felt I was sufficiently aware of the issues related to water contamination.
1.2 Prior to reading the article on floodwater contamination, I felt I was sufficiently aware of the issue.
1.3 Prior to reading the article on forever chemicals in drinking water in New Orleans, I felt I was sufficiently aware of the issue.
1.4 Prior to reading the article on the Flint water crisis, I felt I was sufficiently aware of the issue.

Section 2: Understanding and Appropriateness of Articles

Please rate how much you agree with each of the following statements related to the articles provided:

2.1 I fully understand the contents of the article on forever chemicals in New Orleans water.
2.2 I fully understand the contents of the article on floodwater contamination in New Orleans.
2.3 I fully understand the contents of the article on Flint water contamination.
2.4 I found the article on forever chemicals in New Orleans water appropriate for the lay public.
2.5 I found the article on floodwater contamination in New Orleans water appropriate for the lay public.
2.6 I found the article on Flint water contamination appropriate for the lay public.

Section 3: Deliberation Activity

Please rate how much you agree with each of the following statements related to the deliberation activity:

3.1 Prior to the deliberation exercise, I had already concluded the option I would adopt regarding water contamination issues.
3.2 The deliberation exercise provided more clarity regarding water contamination issues.
3.3 As a result of participating in the deliberations, I learned other points of view related to water contamination.
3.4 The decision regarding the option I favored was formed during the deliberation.
3.5 The decision regarding the option I favored was changed during the deliberation.
3.6 After participating in the deliberation, I am still undecided regarding the option I will favor.

Section 4: Video Presentation Assignment

You will be required to develop a 3–5 minute video presentation that favors one of three options to address water contamination. Please rate your agreement with the following statements:

4.1 I feel confident that I can present an argument for the option I favor in addressing water contamination.
4.2 I feel confident that I can make an effective presentation for the option I favor.
4.3 I understand the technology required to develop the video presentation.
4.4 I feel confident that I can make an effective presentation that can be understood by a lay audience.
4.5 My participation in this class has informed my understanding of water contamination issues.
4.6 My participation in this class has made me a better communicator of science to the public.
4.7 I am uncomfortable with this type of assignment for a chemistry class.
4.8 These types of assignments should be used in my other science classes.

Section 5: Instructor Evaluation

Please indicate your level of agreement with the following statements regarding your instructor:

5.1 The instructor is friendly.
5.2 The instructor establishes a climate conducive to learning.
5.3 The instructor is enthusiastic.
5.4 The instructor provides good examples of concepts.
5.5 The instructor is helpful when students are confused.
5.6 The instructor is open to other viewpoints and ideas.

Section 6: Student Experience

Please indicate your level of agreement with the following statements about your experience:

6.1 Students discuss one another’s ideas.
6.2 Students feel free to disagree with the instructor.
6.3 Students volunteer their own opinions.
6.4 The instructor acknowledges when students have done a particularly good job.

Section 7: Interest and Career Considerations

7.1 What is your current level of interest in chemistry?
7.2 What is your level of consideration for a career that requires communicating science to the public?
7.3 What is your level of consideration for a career that requires communicating science to the public? *(duplicate—consider removing or revising)*
7.4 What is your current level of confidence in communicating science to non-scientists in a private setting?

Section 8: Demographics and Feedback

8.1 Please state your current major.
8.2 Please provide any additional comments related to the deliberation assignment on water contamination.

Optional (Recommended for your study)

Add a Likert scale at the top:
Strongly Disagree | Disagree | Neutral | Agree | Strongly Agree
