# Supplementary material for: Enhancing Science Communication through Deliberative Discussions on Water Contamination in a General Chemistry Lab at an HBCU
Source: J Chem Educ. 2026 Jul 21;103(8):4690–4. doi: 10.1021/acs.jchemed.5c01446 (PMC13472131; doi:10.1021/acs.jchemed.5c01446)
Supplement: Supplementary file 3 [file ed5c01446_si_003.pdf]

Enhancing Science Communication through Deliberative Discussions on Water Contamination in a General Chemistry Lab at an HBCU

Navneet Goyal[1*], Margo N. Montgomery-Richardson[1], Abha Verma[1], Siddieg O Elsiddieg[1], and Jayalakshmi Sridhar[1]
Department of Chemistry, Xavier University of Louisiana, New Orleans, LA-70124, USA.

Keywords: Undergraduate, General chemistry laboratory, Water contamination, Policy, Data Discussion

With a total of 40 questions, along with 5-point Likert-scale answers, this dataset contains multiple survey questions and participant responses related to science communication, deliberation, career interest, confidence, educational background, and general chemistry education. These were further divided into 6 sections. Data on participants' current majors and additional qualitative comments were also collected. The qualitative feedback collected in these questions provides valuable context for the quantitative data.

(Demographics)

Of the 48 students enrolled in two separate sections (24 students each) of the Spring 2024 General Chemistry Teaching Laboratory course, 36 students responded to the survey, which was completed within one week after the deliberation activity. In comparison, only 4 students participated in the survey during Spring 2023; therefore, the present analysis focuses on the Spring 2024 data.

Themes of "supportive faculty" and "engaging labs" were frequently mentioned, highlighting key factors contributing to students' positive experiences. Conversely, challenges such as "limited resources", “financial constraints”, or "time constraints" were also noted, suggesting further opportunities for improvement.

The data also offers insights into potential shifts in attitudes or perceptions over time. For example, cross-referencing survey completion dates with responses related to confidence and career interest reveals possible seasonal or cohort-based trends. If students had been surveyed at the end of the course rather than after a single lab activity, they might have reported higher levels of confidence, suggesting a cumulative benefit from both the activity and the overall course design. This could be a key point in advocating for the adoption of similar pedagogical models.

Responses to questions about career consideration in science (Questions 8 and 9) indicated a strong inclination toward STEM careers, though levels of interest vary. By comparing these responses with demographic data of major fields of study, it was possible to identify the Chemistry Pre-Med students and the Psychology Pre-Med majors, who exhibit the highest interest.

Table S1: Comparing Student Majors' answers to "What is your level of consideration for a career that requires communicating science to the public?" where Very Low interest =1

and Very High interest =5. The average interest value was taken if there was more than one student in a particular major. Total responses=36, Overall average (or interest out of 5) = 3.28.

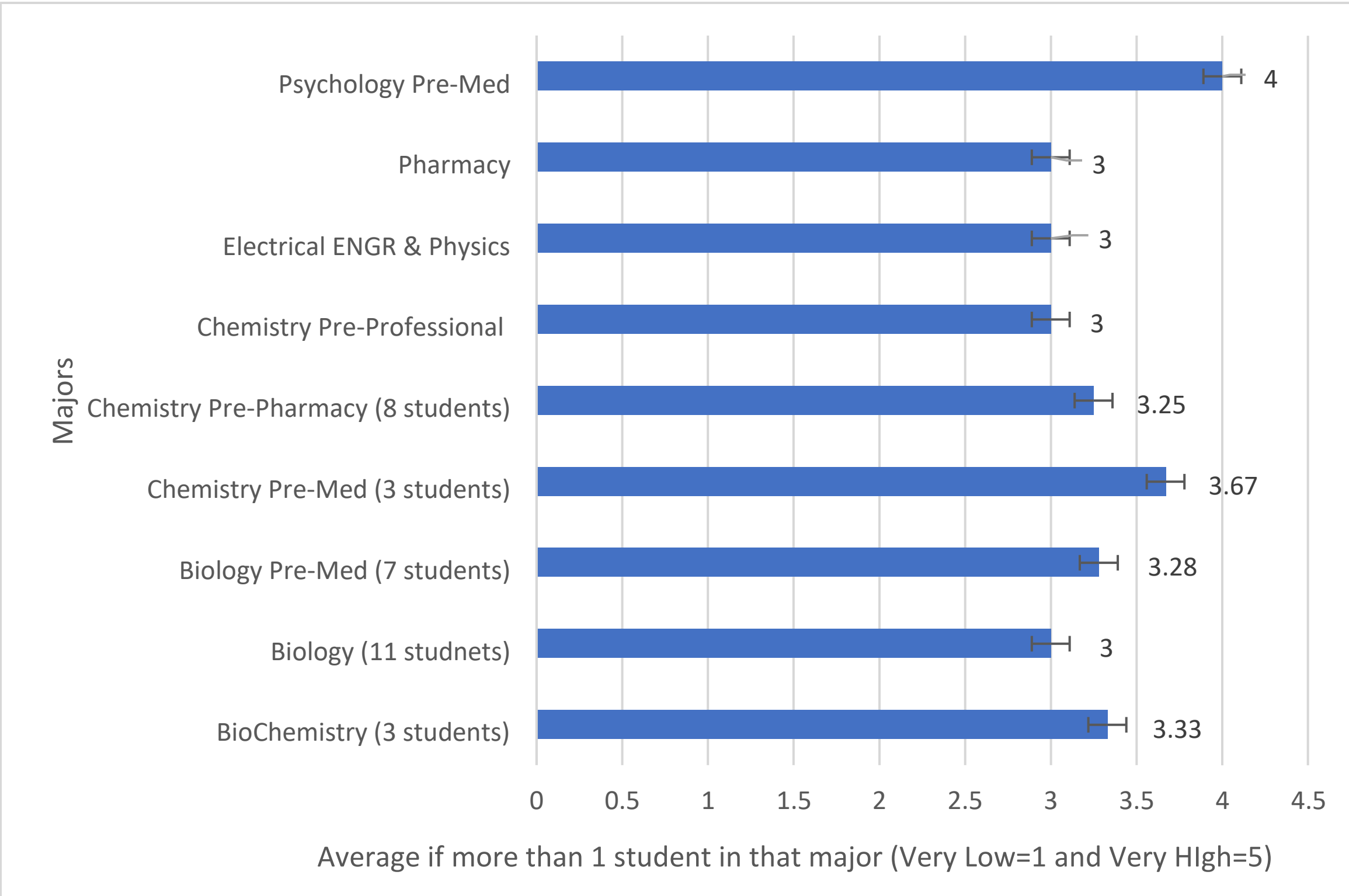

These findings can inform future recruitment strategies for underrepresented minority students. To achieve greater equity across STEM disciplines, efforts should focus on students who express high interest in STEM careers but only moderate confidence in their abilities.

The survey responses reveal notable insights into student attitudes toward science communication and their aspirations for STEM careers. For instance, almost 56% of responding participants reported "High" or "Very High" interest in pursuing science- related careers, as indicated in the aggregated responses to the question, “Currently, what is your level of interest in Chemistry?” This data indicates the effectiveness of the current curriculum in fostering career-oriented motivation among underrepresented minority students. However, the variation in confidence levels, where some participants indicated "Moderate" or "Low" confidence in their scientific abilities, suggests potential areas for intervention, such as targeted mentorship programs or skill-building workshops.

Table S2: Summary of how many students changed from one decision to another.

**Count of Participants**	**The decision regarding the option I favored was changed during the deliberation.**					
**Prior to the deliberation exercise, I had already concluded the option I would adopt in relation to water contamination issues.**	Strongly Disagree	Somewhat Disagree	Neither agree nor disagree	Somewhat Agree	Strongly Agree	**Grand Total**
Strongly Disagree		1	1	1		**3**
Somewhat Disagree		5		3		**8**
Neither agree nor disagree	2	1	7			**10**
Somewhat Agree	1	3	1	5	1	**11**
Strongly Agree				2	2	**4**
**Grand Total**	**3**	**10**	**9**	**11**	**3**	**36**

Further analysis of the Likert-scale responses to questions about science communication and deliberation provides deeper insights into student engagement and the perceived impact of the program. For instance, aggregated data from questions such as "Please state your level of agreement related to confidence in science communication" can highlight how confident students feel when presenting or discussing scientific concepts. Preliminary inspection of these responses reveals a trend toward moderate confidence, indicating room for targeted interventions such as more opportunities for publicspeaking or peer-led workshops.

Table S3: Summary of how many students could not finalize their opinion after deliberation.

**Count of Participants**	**After participating in the deliberation, I am still undecided regarding the option I will favor.**					
**Prior to the deliberation exercise, I had already concluded the**	Strongly Disagree	Somewhat Disagree	Neither agree nor disagree	Somewhat Agree	Strongly Agree	**Grand Total**

**option I would adopt in relation to water contamination issues.**						
Strongly Disagree	1	1		1		**3**
Somewhat Disagree	2	1	3	2		**8**
Neither agree nor disagree	2	1	6	1		**10**
Somewhat Agree	3	1	4	3		**11**
Strongly Agree		2			2	**4**
**Grand Total**	**8**	**6**	**13**	**7**	**2**	**36**

The table S3 shows that only **5.6%** (2 out of 36 students) **strongly agreed** that they were unable to follow an option they favored, while **19.4% somewhat agreed**. The majority of students felt confident in their ability to make decisions following the deliberation activity, suggesting that the deliberation process is effective in empowering students to make informed choices.

Table S4: Summary of a few questions based on student self-rating

**Questions**	**Strongly Disagree**	**Somewhat Disagree**	**Neither agree nor disagree**	**Somewhat Agree**	**Strongly Agree**
I feel confident that I can make an effective presentation for the option I favor that can be understood by a lay audience.	1	5	8	11	11
I feel that my participation in this class has informed my understanding of the water contamination issue.	1	2	8	16	9
I feel that my participation in this class has made me a	1	2	10	16	7

better communicator of science to the public.					
I am uncomfortable with this type of assignment for a chemistry class.	3	9	10	8	6
I think that these types of assignments should be used for my other science classes.	1	5	6	12	11
What is your level of consideration for a career that requires communicating science to the public?	1	2	21	8	4
Currently, what is your level of confidence in communicating science to non-scientists in a private setting?	1	4	26	4	1

A majority of students (22 out of 36) **somewhat agree or strongly agree** that they feel confident in making an effective presentation for a lay audience, while a noticeable portion (14 out of 36) still lack full confidence. This indicates that while many feel prepared after the one deliberation session, some may still need additional support in science communication.

Regarding the understanding of water contamination, only **3 students** disagreed, indicating that the class was largely successful in achieving this learning outcome. A significant number of students (25 out of 36) **somewhat agree or strongly agree** that their class participation improved their understanding of water contamination.

Most students (23 out of of 36) feel that the class has improved their ability to communicate science to the public. A smaller group (10 students) remained neutral, which suggests that while the majority saw a benefit, some may not have perceived a strong impact on their communication skills.

Responses are more evenly distributed on the question asking about discomfort with this type of assignment, with **12 students somewhat or strongly disagreeing** that they are uncomfortable, while **14 students are neutral or agree** with the statement. This indicates a mixed reaction—some students are open to this type of assignment, while others may feel uneasy about integrating non-traditional coursework into a chemistry class.

A majority (23 out of 36) support incorporating similar assignments in other science classes. However, **6 students neither agree nor disagree**, and **6 students disagree**, showing that while many find value in these assignments, some may not see them as universally applicable.

Overall, the data strongly suggests that the assignment enhanced students' understanding of water contamination and improved their ability to communicate science.

The Role of Science Communication:

The survey questions focusing on science communication reveal another dimension of student development. Confidence in communicating scientific concepts is crucial for academic success and career readiness. By analyzing responses to these questions, we can assess whether current training activity effectively prepares students for real-world applications, such as presenting at conferences or collaborating in research settings.

For example, as shown in Table 4, since a majority of students report "Moderate" confidence in considering a career that requires communicating science to the public, this could signal the need for additional workshops, practice sessions, or opportunities for students to present their work publicly.

Conclusions:

The data strongly suggests that the assignment enhanced students' understanding of water contamination and improved their ability to communicate science, indicating a positive impact on learning and communication. The data also shows that there are gaps in confidence levels. While many students feel confident in presenting, a noticeable portion (14 out of 36) still lack full confidence. Moreover, the study suggests that there are mixed reactions to the assignment format. While many students are comfortable with this type of assignment and want it in other science courses, some remain uncertain or uncomfortable.
